# Endogenous Fluorescent Proteins in the Mucus of an Intertidal Polychaeta: Clues for Biotechnology

**DOI:** 10.3390/md20040224

**Published:** 2022-03-25

**Authors:** Ana P. Rodrigo, Ana Lopes, Ricardo Pereira, Sandra I. Anjo, Bruno Manadas, Ana R. Grosso, Pedro V. Baptista, Alexandra R. Fernandes, Pedro M. Costa

**Affiliations:** 1 UCIBIO—Applied Molecular Biosciences Unit, Department of Life Sciences, NOVA School of Science and Technology, Universidade NOVA de Lisboa, 2829-516 Caparica, Portugal; arc.lopes@campus.fct.unl.pt (A.L.); rb.pereira@campus.fct.unl.pt (R.P.); ar.grosso@fct.unl.pt (A.R.G.); pmvb@fct.unl.pt (P.V.B.); ma.fernandes@fct.unl.pt (A.R.F.); 2 Associate Laboratory i4HB, Institute for Health and Bioeconomy, NOVA School of Science and Technology, Universidade NOVA de Lisboa, 2829-516 Caparica, Portugal; 3 Center for Neuroscience and Cell Biology, University of Coimbra, Parque Tecnológico de Cantanhede, Núcleo 04, Lote 8, 3060-197 Cantanhede, Portugal; sandra.isabel.anjo@gmail.com (S.I.A.); bmanadas@gmail.com (B.M.)

**Keywords:** fluorescence, mucosecretions, UV light, ubiquitin conjugates, marine Annelida, redox status, proteome, mass spectrometry, poisonous secretions, transcriptomics

## Abstract

The vast ocean holds many unexplored organisms with unique adaptive features that enable them to thrive in their environment. The secretion of fluorescent proteins is one of them, with reports on the presence of such compounds in marine annelids being scarce. The intertidal *Eulalia* sp. is an example. The worm secretes copious amounts of mucus, that when purified and concentrated extracts, yield strong fluorescence under UV light. Emission has two main maxima, at 400 nm and at 500 nm, with the latter responsible for the blue–greenish fluorescence. Combining proteomics and transcriptomics techniques, we identified ubiquitin, peroxiredoxin, and 14-3-3 protein as key elements in the mucus. Fluorescence was found to be mainly modulated by redox status and pH, being consistently upheld in extracts prepared in Tris-HCl buffer with reducing agent at pH 7 and excited at 330 nm. One of the proteins associated with the fluorescent signal was localized in secretory cells in the pharynx. The results indicate that the secretion of fluorescent proteinaceous complexes can be an important defense against UV for this dweller. Additionally, the internalization of fluorescent complexes by ovarian cancer cells and modulation of fluorescence of redox status bears important considerations for biotechnological application of mucus components as markers.

## 1. Introduction

Fluorescence was first reported in animals in the 19th century—more specifically, in the feathers of some species as a mean to lure females during mating season. From this point onward, reports of luminescence sprang up from the whole animal kingdom, a concept that precludes fluorescence, bioluminescence, and chemiluminescence (see Lagorio et al. [1] for further details on historical aspects). Bioluminescence has arguably received most of the attention (recall the luciferin–luciferase system) since the discovery of the green fluorescent protein (GFP), a photoprotein that yields both bioluminescence and fluorescence and was first isolated from the hydrozoan medusa *Aequorea victoria* [2]. Since then, the exploitation of luminescent substances of marine origins gained momentum, in large part pushed by the biotechnological applications of GFP in its natural and recombinant forms (see Tsien [3] for a review).

Animal bioluminescence, chemiluminescence, or fluorescence invariably relates to feeding, mating, or defense mechanisms [4]. Due to its diversity of habitats and concomitant biodiversity, the marine environment has revealed an extraordinary span of luminescent organisms. Deep-sea fish, in particular, are well-known to explore endogenous- or symbiotic-driven bioluminescence, whereas those in shallower waters exploit the restricted spectra of sunlight penetrating the depths by producing fluorescent patterns to reduce or enhance visual contrast [5]. Although less explored, marine invertebrates may hold even more diverse luminescent adaptations to their environment. Indeed, among the Cnidaria alone, several groups besides hydrozoans were found to have GFP analogues, such as the Anthozoa [1]. Organisms from this class possess fluorescent proteins that regulate the availability of sunlight to polyps [6]. Fluorescence was also detected in Siphonophores, which has been attributed to the presence of porphyrins in their tentacles that are bioluminescent and red fluorescent, a feature that is believed to lure fish [7,8]. Among marine invertebrates, Cnidaria is not only the phylum with the most identified fluorescent substances but it is also the best-studied invertebrate group for biotechnological applications, from food to insecticides [9]. Still, in Arthropoda, copepods from the family Pontellidae are known to possess GFP-like proteins for recognition [10]. In turn, cephalochordate GFP-like proteins are thought to play a role in photoreception and photoprotection against ultraviolet (UV) and blue light [11]. Fluorescence has also been attributed to microorganisms, such as bacteria and algae, living in symbiosis with marine organisms. For example, algae living in symbiosis with corals facilitate light availability and may have a photoprotective role [1]. Fluorescence resulting from symbiosis in marine invertebrates has been used to understand their interactions in novel ways and use their fluorescence in techniques such as live cell analysis [12].

Information on fluorescent substances in Polychaeta are scarce [13] despite being one of the most abundant and diversified taxa that occupy every habitat from deep-sea hydrothermal vents to the intertidal [14]. The first reports began with *Chaetopterus*, whose chaetopterin pigments was found to be fluorescent (see, for instance, Nicol [15] and Kennedy and Nicol [16]). Fluorescent photoproteins have also been found in the scales of scale worms, Polynoidae [17]. *Eulalia* sp., a green-colored predator polychaete of the rocky intertidal, has recently been investigated for its porphyrinoid pigments, seemingly associated with UV absorption as a protective measure while foraging [18]. Even though these pigments are not fluorescent, the toxin-bearing mucus secreted by the worm [19] possesses unknown fluorescent proteinaceous substances that strongly emit blue–green light when excited with UV light [20]. We hypothesize that the presence of fluorescent compounds in the mucus is an adaptation to provide the worm with an additional defense against exposure to daylight. The aim of this work is thus a contribution to the identification of proteinaceous compounds responsible for the fluorescence of the mucus secreted by *Eulalia* sp. and the main exogenous variables that modulate emission.

## 2. Results

### 2.1. Molecular Characterization

**Figure 1:**
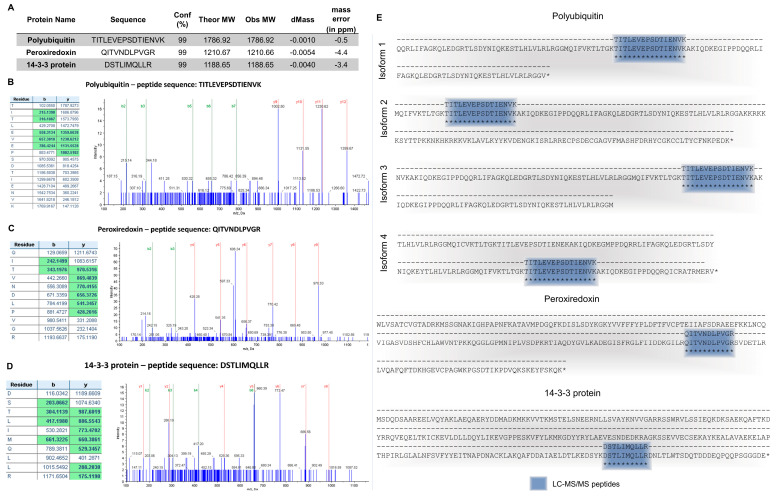
Identification of proteins responsible for the fluorescence of the mucus secreted by the marine Polychaeta *Eulalia* sp. (**A**) List of the peptides’ sequences (identified using the ProteinPilot software) and the respective confidence (conf) according to Paragon™ Algorithm, theoretical and observed molecular masses (Theor MW and Obs MW, respectively), the mass difference (dMass), and mass error in ppm. (**B**) MS/MS spectrum from the peptide TITLEVEPSDTIENVK identified with 99% confidence. (**C**) MS/MS spectrum from the peptide QITVNDLPVGR identified with 99% confidence. (**D**) MS/MS spectrum from the peptide DSTLIMQLLR identified with 99% confidence. The fragment ions that were correctly assigned to the theoretical spectrum are highlighted in green on the left panels and indicated at the MS/MS spectra (right panels). The existence of a sequence of at least four consecutive ions improves the confidence in the identification of these peptides. (**E**) Alignment of peptides obtained by LC-MS/MS (in blue) with the translated mRNAs obtained from whole-transcriptome assembly following RNA-Seq. Similarities are indicated by *.

Purified protein extracts from mucus in unstained SDS-PAGE gels yielded strong blue–green fluorescence when subjected to UV transillumination (Figure S1A). Gels revealed two almost joint main fluorescent bands (with 48 kDa approximately, Figure S1B) with a smear of dimmer bands corresponding to lower molecular weight peptides. The proteinaceous band was visualized by transillumination with visible light after Coomassie and silver staining in SDS-PAGE gels. However, no fluorescent or stained bands were detected in native gels (Figure S1). The fluorescent bands that were excised from SDS-PAGE gels showed—by matching MS/MS results with sequences obtained by analyzing *Eulalia*’s transcriptome—the presence of three peptides and proteins (Figure 1C, Table S1): polyubiquitin, peroxiredoxin, and 14-3-3. Both polyubiquitin and 14-3-3 proteins have very characteristic domains (ubiquitin-like domain and 14-3-3-beta-zeta domain). Peroxiredoxin (Prx) was cross-validated through the identification of a conserved domain belonging to the 2-Cys Prx subfamily. Polyubiquitin was present in four distinct isoforms (Figure 1C).

### 2.2. Modulation of Absorption and Fluorescence

**Figure 2:**
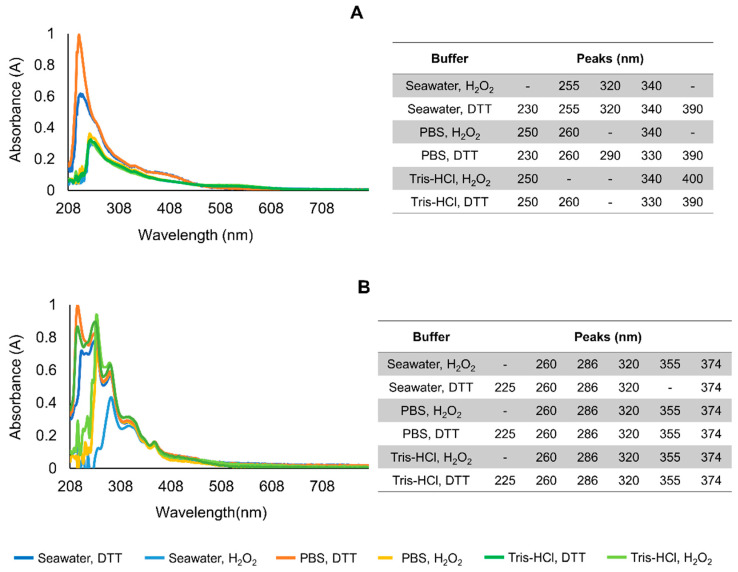
Averaged absorbance spectra of mucus samples in different buffers (seawater, PBS, and Tris-HCl pH 7) and redox treatments (H_2_O_2_ and DTT) and the respective peaks. (**A**) Crude mucus samples. (**B**) Purified mucus samples. Experiments were performed in triplicate with independent extracts. Concentration of extracts was normalized to 1 mg total protein, mL^−1^.

Differences were found between the absorption spectra of crude and purified extracts excited with light within the UV-A range (200–800 nm) (Figure 2). Crude mucus samples yielded a single maximum at 230–260 nm depending on the buffer, whereas purified mucus yielded multiple absorption peaks, namely, at 225, 260, and 286 nm. The absorption spectra of crude samples were similar between treatments with different buffers/media and redox agents (H_2_O_2_ or DTT), while the spectra of purified samples had great variability at shorter wavelengths (200 to 300 nm). This variation was mostly redox-agent-dependent.

**Figure 3:**
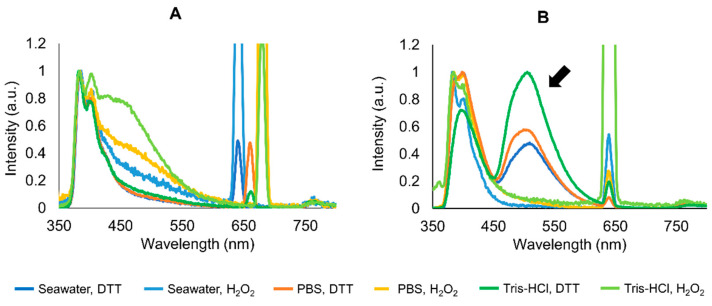
Averaged and normalized emission spectra of crude (**A**) and purified by ultrafiltration (**B**) mucus samples in different media buffers (seawater, PBS, and Tris-HCl buffers pH 7) and redox treatments (H_2_O_2_ and DTT). Samples were excited at 320–340 nm, corresponding to absorbance maxima. Arrow is indicative of visible fluorescent maxima. Experiments were performed in triplicate (three independent purified extracts). Concentration of extracts was normalized to 1 mg total protein, mL^−1^.

Each sample was then excited with light corresponding to the respective absorption maxima. The highest emission intensity in crude mucus samples was obtained in oxidized spectra excited at 260 nm (Table S2), but at around 330 nm, the spectra were more consistent for both crude (Figure S2) and purified mucus samples (Table S3, Figure S3). Overall, the emission spectra were more stable at around 330 nm than other excitation wavelengths for crude and purified samples (Figure 3). The emission maxima ≈ 400 nm was consistent, whereas maxima at 505–510 nm, responsible for blue–greenish fluorescence, was only present in purified extracts (Figure 3B, indicated by the arrow). Moreover, in purified samples, redox status was a key factor for the presence of the maxima at ≈500 nm in samples treated with DTT (Figure S4). This peak was consistent in all the replicates (Figure S3). Overall, reduced samples in Tris-HCl yielded higher fluorescence (Figure S4 and Figure 3B).

**Figure 4:**
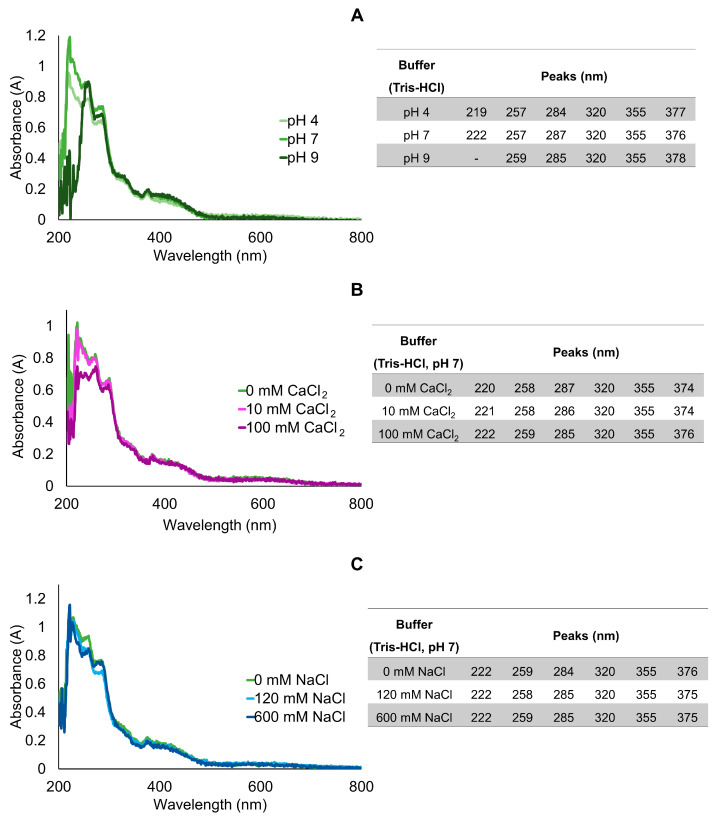
Averaged absorbance spectra of purified mucus samples in Tris-HCl supplemented with 20 mM DTT buffer. (**A**) Different pH (4, 7, and 9). (**B**,**C**) Samples in Tris-HCl pH 7 complemented with different concentrations of CaCl_2_ and NaCl, respectively. Experiments were performed in triplicate (three independent purified extracts). Concentration of extracts was normalized to 1 mg total protein, mL^−1^.

The combined effects of modulating pH, calcium, and salinity on absorption spectra are present in Figure 4. The absorption spectra of the purified extracts in Tris-HCl buffer (supplemented with DTT) with different pH showed little change, albeit a trend to increase absorption at lower wavelengths with decreasing pH, which led to the absence of a peak at ≈220 nm at pH 9 (Figure 4A). As such, due to the more consistent results provided by Tris-HCl pH 7, this medium was chosen for subsequent analyses with different concentrations of CaCl_2_ and NaCl. Still, purified extracts in Tris-HCl buffer pH 7 with different concentrations of Ca (as CaCl_2_) and salt (as NaCl) yielded negligible differences between absorption spectra (Figure 4B,C, respectively).

**Figure 5:**
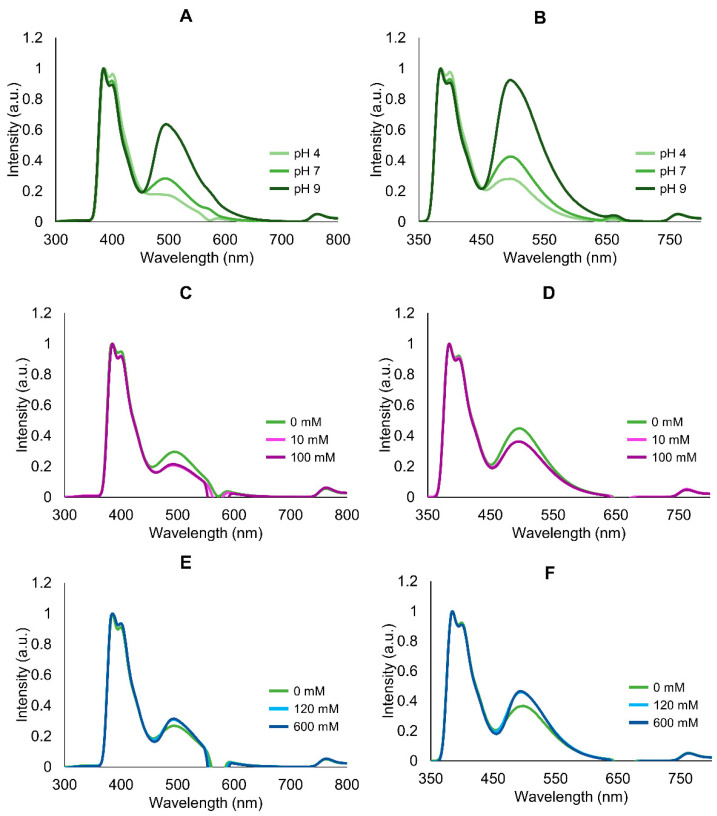
Averaged and normalized emission spectra of purified mucus samples excited at 285 nm and 330 nm. Samples comprising protein extracts in Tris-HCl buffer supplemented with 20 mM DTT, tested with different pH, salinity, and Ca concentration. (**A**,**B**) pH 4; 7 and 9, excited at 285 and 330 nm, respectively. (**C**,**D**) Calcium (as CaCl_2_), 0; 10 and 100 mM, excited at 285 and 330 nm, respectively (pH 7). (**E**,**F**) NaCl, 0; 120 and 600 mM, excited at 285 and 330 nm, respectively (pH 7). Experiments were performed in triplicate (three independent purified extracts). Concentration of extracts was normalized to 1 mg total protein, mL^−1^.

**Figure 6:**
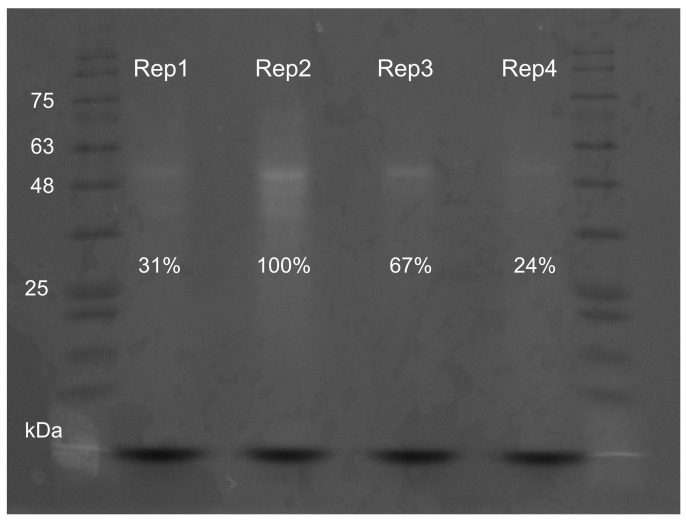
SDS-PAGE of purified protein extracts from *Eulalia*’s mucosecretions, visualized by trans-UV (unstained). Four different samples (identified as replicates 1 through 4), were prepared in Tris-HCl buffer (pH 7), supplemented with 20 mM DTT. The amount of total protein was normalized to 1 mg·mL^−1^. Relative fluorescence intensity is expressed as a percentage.

Increasing buffer pH led to an overall increase in fluorescence intensity, with a peak at 496 nm corresponding to green–blueish light, higher after excitation at 330 nm (see Figure 5A,B and Figure S5 for absolute values). However, despite strongest emission, extracts in Tris-HCl pH 9 yielded higher inter-replicate variation regardless of excitation wavelength (Figure S6). For these reasons, previously, Tris-HCl pH 7 was selected as medium for the subsequent tests. In accordance with the reduced variation in absorption spectra, the emission spectra of samples treated with different concentrations of Ca and NaCl yielded reduced variation. Calcium caused a small decrease in emission intensity regardless of concentration, compared with control (Figure 5C–F). Similarly, salinity did not produce an obvious effect in emission (Figure 5E,F). Both treatments yielded, however, noticeable inter-replicate variation (Figures S7 and S8) that was verified by SDS-PAGE as well (Figure 6), revealing a variation in emission intensity down to 24% among replicates normalized to 1 mg total protein, mL^−1^.

### 2.3. Histological Localization of the Fluorescent Secretions

**Figure 7:**
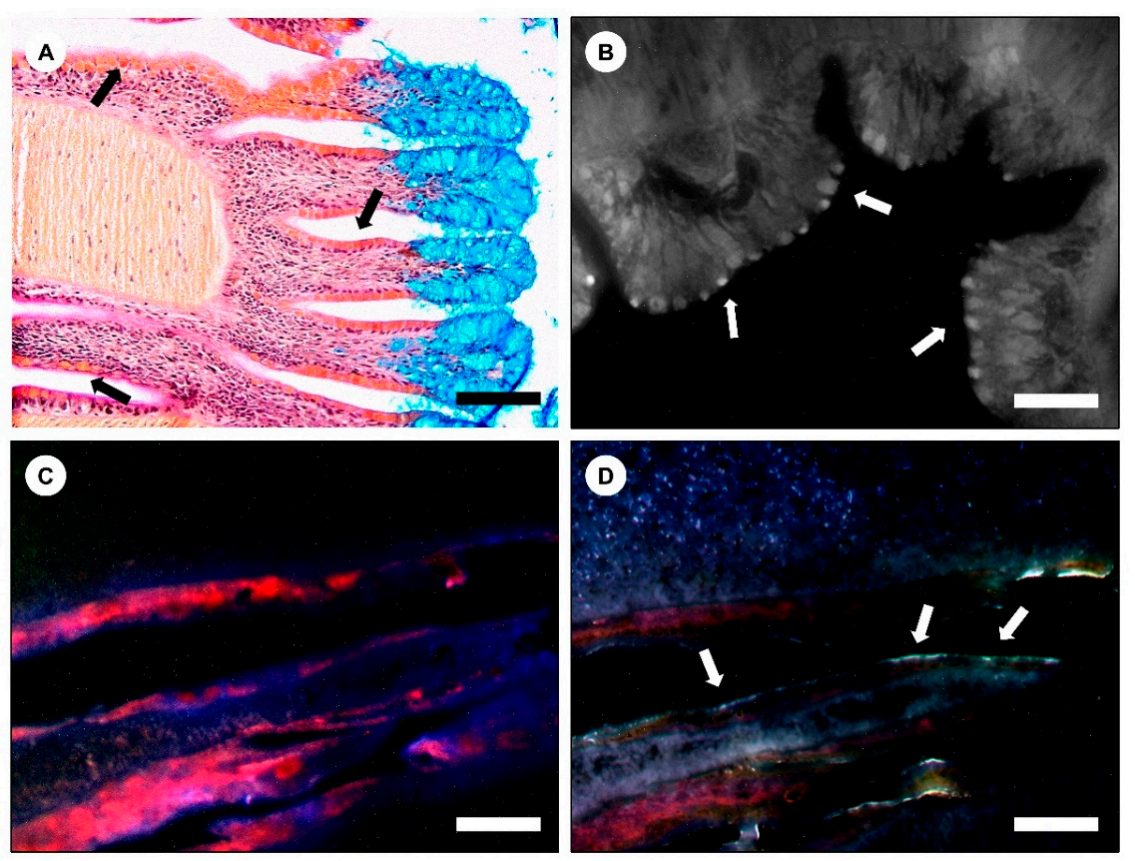
Localization of mucosecretions in histological sections of *Eulalias*’s proboscis through their fluorescent signal. (**A**) Histological section (fixed with glutaraldehyde) stained with a tetrachrome stain, showing the tentacles in the anterior section of the pharynx. Arrows are indicative of secretory calyx (serous) cells. Scale bar: 50 µm. (**B**) Histological section fixed with glutaraldehyde and treated with DTT (20 mM). Arrows show fluorescent secretory calyx cells. Scale bar: 25 µm. (**C**) Longitudinal cryosection treated with 2% H_2_O_2_ (*v*/*v*). Scale bar: 50 µm. (**D**) Longitudinal cryosection treated with DTT (20 mM). Arrows are indicative of fluorescence signal lining the inner epithelium of the pharynx. Scale bars: 50 µm.

**Figure 8:**
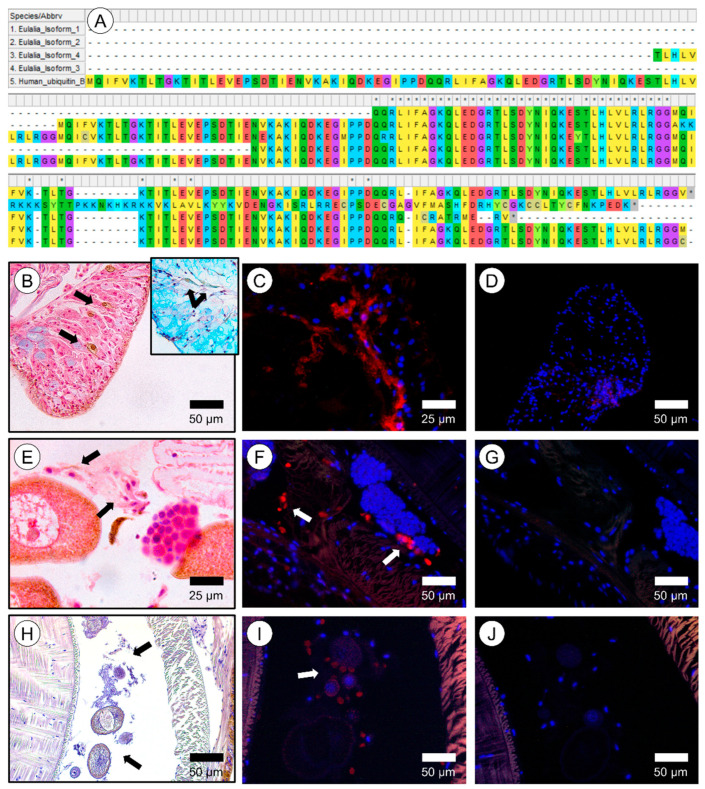
Immunohistochemical localization of ubiquitin B in *Eulalia*. (**A**) Alignment of peptides from MS/MS on fluorescent SDS-PAGE gel bands, RNAseq sequences with positive matches to ubiquitin, and the human ubiquitin B sequence against which the polyclonal antibody was used. The antibody was chosen based on similarity with *Eulalia* ubiquitin isoform 3. (**B**) Paraffin section across a parapodium of the worm fixed with Zenker and stained with HE (hematoxylin and eosin). Hemocytes are identified by the arrows. Inset: Paraffin section fixed with glutaraldehyde and stained with TC (tetrachrome stain) showing hepatocytes naturally colored by green pigments. (**C**) Compositive of a cryopreserved section of a parapodia marked for ubiquitin B. (**D**) Negative control of cryopreserved section of a parapodia. (**E**) Paraffin section fixed with Zenker and stained with HE; arrows are indicative of stem cells. (**F**) Composite of a paraffin section of the ventral lateral section marked for ubiquitin B. Arrows indicate the stem cells marked by the antibody. (**G**) Negative control of cryopreserved section of the ventral lateral area, close to the parapodia. (**H**) Paraffin section fixed with glutaraldehyde and stained with HE, showing the female gametes in the celomic cavity. (**I**) Composite of celomic cavity with positive signal for ubiquitin B (arrow). (**J**) Negative control of cryopreserved section of the celomic cavity.

Fluorescent secretions were histologically localized in secretory cells in the pharynx at the proboscis after treatment with DTT (unstained) and excitation with UV light (Figure 7A,B). Fluorescent secretions were also revealed in cryosections treated with DTT and left unstained (Figure 7C,D). In this case, the fluorescent signal was detected in cells as well as in the adjacent mucus layer. The secretion of one of the isoforms (isoform 3, recall Figure 8A) of polyubiquitin was localized immunohistochemically (Figure 8). The presence of this isoform was noticed mainly in parapodia and in some defense cells, i.e., coelomocytes (Figure 8B,C). Additionally, polyubiquitin was also detected in initial stage oocytes in the oocyte cuticle (Figure 8E–J).

### 2.4. Internalization by A2780 Cells

**Figure 9:**
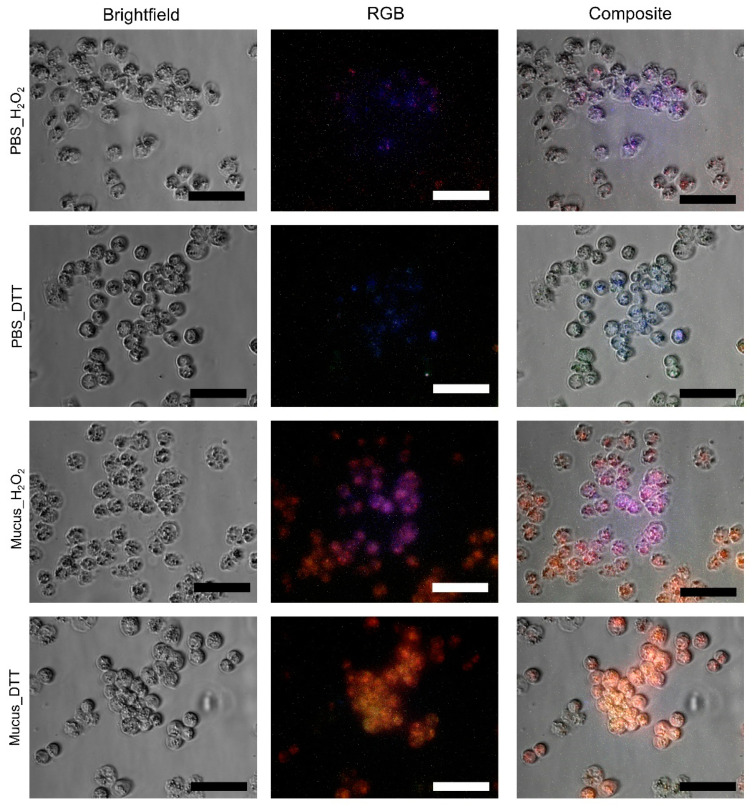
Internalization assay for purified mucosubstances onto the human ovarian cancer cell line A2780. Cells were incubated during 3 h with purified mucus extract prepared with PBS (pH 7.4) and the respective control (PBS only). Either experimental condition was subjected to reduced or oxidizing agents (DTT and H_2_O_2_). Composite images were produced by overlapping red, green, and blue channels with brightfield images. Scale bars: 25 µm.

After 3 h exposure, we observed increased fluorescent signal within A2780 cells, which was more intense in cells treated with DTT (Figure 9). Residual fluorescence could be noticed following treatment with H_2_O_2_ and PBS. The fluorescent signal increase in cells treated with DTT in a time-responsive manner, as early as 1 h and after 6 h, was evident in all cells. Conversely, only after 3 h of exposure could the signal be found in cells treated with H_2_O_2_ (Figure S9). The mucus gave a reddish fluorescence to cells after internalization, whereas in controls, the cells presented a bluish fluorescence. The mucus cytotoxicity was assessed in the same cells in previous works [21].

## 3. Discussion

The mucus secreted by *Eulalia* bears a proteinaceous complex that yields strong fluorescence when excited with UV light. Emission, within the range of the blue–green spectrum, is significantly modulated by redox status, increasing when the mixture is reduced and decreasing upon oxidation, reversibly, thus acting like a biochemical switch. Another important factor is pH, as emission tends to increase with alkalinity. However, normal physiological pH seems to offer a good compromise between emission intensity and intersample consistency. The results also indicate that, aside from the shift in redox status, the vehicle (buffer) is versatile, with the compounds retaining their basic properties in both seawater and physiological media, as well as in Tris-HCl, one of the buffers of choice to extract proteinaceous substances in the laboratory, which may indicate potential biotechnological interest. Altogether, these findings suggest that the worm actively secretes these compounds to the mucus, remaining fluorescent in seawater until oxidation, with salinity and Ca concentrations rendering little or null impact on emission. The fluorescent complex likely contributes to protection against UV light by absorbing UV radiation and releasing energy in the form of visible light, holding antioxidant properties as well. These properties confer advantages to an intertidal diurnal forager such as *Eulalia*, who actively roams the rocky intertidal in search of prey. Other functions for the fluorescence, such as communication and warning, remain speculative at this stage. Although, it must be noted that most reports on luminescence in marine invertebrates focus on bioluminescence. Indeed, there is scant information on the secretion of fluorescent substances by Polychaeta or Annelida in general (see Rodrigo and Costa [13] for further details). Nevertheless, the ecological purposes of bioluminescence and fluorescence, such as warning or defense, can be similar [4]. In the particular case of *Eulalia*, the uncanny bright green pigmentation provided by the accumulation of porphyrinoid pigments, which absorb UV light, already indicate that these animals are particularly well-fit to rove through the rocky intertidal [18]. On the other hand, the copious secretion of mucus by the worm has multiple roles, namely, lubrication during locomotion, protection against desiccation, and toxin delivery [22], to which we may now add important functions against UV and oxidation.

The emission spectra of purified *Eulalia* mucus extracts present some similarities with other organisms secreting green, fluorescent proteinaceous compounds, such as a maximum at around 500 nm, from the flowers of *Achillea millefolium* and the roots of *Raphanus sativus* [23] to wild-type GFP from *Aequorea* [3]. Such similarities may indeed confirm the proteinaceous nature of *Eulalia* fluorochromes. Tsien [3] also noticed variations of the emission peak with the excitation wavelength, indicative of, at least, two chemically distinct species interfering with fluorescence (see Table S7 for averaged maxima). We recorded a similar occurrent in *Eulalia*, as the fluorescent peak (around 500 nm) shows small variations that depend mostly on excitation wavelength. This is no surprise in *Eulalia* due to the presence of several proteins in the extract [19], three of which were positively identified (ubiquitin, 14-3-3 protein, and peroxiredoxin) using RNA-Seq and MS/MS, that target mRNAs and proteins, respectively—However, the exact nature of the fluorescent proteins or peptides or their mixtures (or even if complexes can be involved) remain, at this stage, elusive.

Ubiquitin is a small c.a. 8.5 kDa regulatory protein (comprised approximately of 76 amino acids) that is well-conserved among eukaryotes. Ubiquitin can be found conjugated with other proteins or in free form as polyubiquitin. When conjugated, it can be linked to lysin residues of target proteins and, in rare cases, conjugated to cysteine or serine residues [24]. There are several ubiquitin-binding domains characterized by the type of proteins they bind to, having associations ranging from zinc finger domain to domains typical of endoplasmic reticulum degradation (see Hicke et al. [25] for a review). Ubiquitin is mostly associated with the function of the proteasome by tagging misfolded or damaged proteins for lysis [26]. Even though there is scant information on the function of ubiquitin in animal secretions, it is found in the venom of some species, such as snakes, as a nontoxic protein with unknown function [27]. In *Eulalia* sp., an isoform homolog to human and mouse ubiquitin B was chiefly identified in defense cells, coelomocytes, or static cells in the parapodia and in the membrane of female gametes (recall Figure 8). This isoform (isoform 3) is the most conserved of the isoforms identified in the fluorescent band. This form is part of the innate immune response, being involved in related cell signaling cascades in humans [28] and other organisms, which explains its presence in *Eulalia* defense cells (recall Figure 8B,C). Even though this isoform was not immunolocalized in the proboscis, its secretion is notorious in the parapodia, which are major players in mucus secretion. We may, therefore, infer a relevant immune function of the mucus and the role of ubiquitin. If indeed associated to fluorescence, polyubiquitin may explain the multiple, slightly different molecular weight bands observed in gels (Figure 1B).

Peroxiredoxins (22–27 kDa) are important antioxidant proteins. These proteins reduce a range of hydroperoxides to protect against reactive oxygen species (ROS). Peroxiredoxins can be found in almost every animal tissue where they can act as peroxide scavenging enzymes, as well as being key players in immunity and as regulators of cell death (see Abbas et al. [29] for a review). They can also be found in the secretion of parasitic nematodes to avoid the host immune response [30]. Their presence in *Eulalia*’s mucus confirms its antioxidant properties and may offer a link to the modulation of fluorescence by changes in redox potential. In turn, 14-3-3 (28–30 kDa) are conserved in most eukaryotic organisms that can bind to several different proteins and, thus, be involved in important cellular processes such as signal transduction, cell-cycle control, apoptosis, and stress response [31]. When found in mucus, 14-3-3 proteins are linked to the immune response of fish and thought to be related with antimicrobial resistance in fish (such as the zebrafish and lumpsucker) and insects [32,33,34].

The association between these three proteins in mucosecretions of Polychaeta as well as their role in fluorescence has not been reported before. However, their presence in mucus secretions has been described in fish, cephalopods, and even in flatworms as part of complex cocktails of substances [33,35,36,37]. Additionally, there are reports of the presence of ubiquitin C and 14-3-3 protein in the integument of the parasitic flatworm *Schistosoma japonicum*, presumably with a role in maintenance of tegument integrity as part of the defense mechanisms against the host response [38]. Curiously, the interactions between ubiquitin and 14-3-3 protein have been identified in tomato plant as being involved in cell-wall-related metabolism and, more specifically, cell-wall building and resistance, degradation of xyloglucan, among other functions [39]. In fact, the connection between 14-3-3 and ubiquitin may have the same protective functions in the cuticle that outlines the inner epithelium of the pharynx of *Eulalia* (recall Figure 2 and Figure 8). If considering only these two proteins (ubiquitin and 14-3-3 protein), their size (48 kDa) is approximately the same as the fluorescent band in gels (≈50 kDa). If indeed forming complexes, these exclude Prx (approximately 22 kDa). It must be noted, however, that ubiquitin can be a chaperon of peroxiredoxins, as demonstrated by the binding abilities of the deubiquitinating enzyme ubiquitin C-terminal hydrolase-L1 (UCH-L1) to 2-Cys Prx in vitro [40]. Nonetheless, their interaction in mucosecretions is unknown. The fact that Prx belongs to a family of peroxidases that bind either thermally or oxidatively to free radicals [29] may explain its influence on redox status of mucus and in its fluorescence. In fact, Prx is ubiquitous in Eumetazoa, including in marine annelids. In *Arenicola marina*, for instance, Prx is mostly present in tissues directly exposed to the external environment, enhancing its importance in the protection against oxidative stress [41]. In fish, their role in mucus as an antioxidative agent has been reported with their part in protection against infections [42].

The fluorescence of ubiquitin and its modulation with redox state and pH has been reported elsewhere. For instance, Noronha et al. [43] found that bovine ubiquitin increased its fluorescence with pH. Curiously, the same pH effect was seen in a GFP-based calcium sensor, a fluorophore very different from ubiquitin, generating two major emission peaks: one around 400 nm and the other around 500 nm [44]. This variation was thus similar to the variation with pH seen in *Eulalia*. In turn, the interference of Ca and Na ions was seemingly negligible on the fluorescence of mucosecretions. There are, however, contradictory reports on the influence of salts on fluorochromes from marine animals. As an example, the fluorescence of hyalin—a fluorescent protein from some echinoderms—has been found to be stabilized by increasing Ca concentrations up to 1 mM CaCl_2_ [45]. On the other hand, the fluorescence of some recombinant GFP is decreased by Ca binding [46]. By increasing the concentrations of NaCl (to 100 mM), the fluorescence of hyalin has been increased by almost 45% [45]. In *Eulalia*, concentrations in the same order of magnitude (120 mM) and even higher (600 mM) did not interfere with fluorescence.

The fluorescent peptides and proteins in the mucus are not secreted by mucocytes but rather, by specialized calyx cells lining the interior of the pharynx that are also responsible for the secretion of toxins, being then conveyed by the mucus [22]. These results indicate that the secretion of fluorescent substances may be linked to toxin secretion and, therefore, to feeding and defense mechanisms [47]. However, the presence of the fluorescent band in the mucus extract is not proportional to the total amount of protein. Indeed, it must be highlighted that there is noticeable interindividual variation in aspects such as color and viscosity of the poisonous fluorescent cocktail secreted by worms (data now shown). Such variation is accompanied by differences in defensive or offensive behavior when the secretions were being harvested (the reader is diverted to the videographic material provided by Cuevas et al. [47]). This interindividual variability shows that the mucus is a far more complex mixture of proteinaceous materials than expected (recall Figure 6). When a physiologically compatible buffer (PBS) is employed as vehicle, these proteins present in the extract can be internalized by human cells and retain their fluorescence, especially if previously treated with a reducing agent. However, internalization shifted the fluorescence signal from blue–greenish to yellow–reddish by undisclosed factors that likely reflect the internal milieu of the target cells (A2780). Some studies attempt to achieve the same results chemically, obtaining a fluorescent signal that is both temperate- and time-dependent to study intracellular dynamics [48]. These changes may be caused by increased temperature (37 °C), the cells’ internal pH, or by cell metabolic activity. In any case, internalization by living cells breaks ground towards application, as it indicates bioreactivity without loss of function, to which we added the uniqueness of modulating redox status as switch. 

The marine Polychaeta *Eulalia* sp. secretes copious amounts of mucus that serve multiple purposes, from lubrication and protection against desiccation to toxin delivery. The secretion of a protein mixture that holds fluorescent properties when exposed to UV light and responds to oxidation suggests that the mucus also contributes to protect this intertidal worm during foraging. Even though the exact chemical nature of the complex of proteins responsible for the fluorescence remains unconfirmed, ubiquitin is seemingly a key player, as well as a peroxiredoxin. The fluorescence seems to be activated by the presence of, at least, these two proteins, rendering one of the most interesting properties of the complex, as it holds the ability to be switched on or off reversibly by reduction or oxidation under physiologically compatible conditions. Altogether, with cellular uptake and the retention of fluorescence in various buffers and with various salts, these properties are indicative of biotechnological potential.

## 4. Materials and Methods

### 4.1. Protein Extraction

*Eulalia* sp. (≈120 mm total length and weighting ≈250 mg each) were hand-collected at rocky intertidal beaches on the west coast of Portugal. Animals were reared in a laboratory in a microcosm environment fitted with natural pebbles and clumps of mussels to provide shelter and feed. Crude mucus samples were harvested noninvasively through mechanic perturbation in sterile and filtered seawater. Each crude sample consisted of a pool of mucus harvested from 50–80 worms. Buffer was immediately added (1:1) to each sample: 0.05 M Tris-HCl pH 7, containing 10% *m*/*v* L-dithiothreitol (DTT) and 1% *v*/*v* protease inhibitor cocktail (Sigma-Aldrich, St. Louis, MO, USA). Samples were then filtered through a cellulose acetate filter (0.22 µm) to remove solid materials and polymerized mucins. Extracts were then subjected to dialysis using 3 kDa Amicon ultrafiltraton tubes (Merck, Kenilworth, NJ, USA) to replace the buffer with experimental media: sterilized seawater, phosphate-buffered saline, or 0.05 M Tris-HCl. Total protein in purified extracts was quantified spectrophotometrically using a Nanodrop 1000 (Thermo Scientific, Waltham, MA, USA). Each replicate contained approximately 7 to 10 mg·mL^−1^ total protein before dilution to normalized concentrations. Samples were stored at −80 °C until further analyses. 

### 4.2. Experimental Design

A tiered battery of experiments was performed to assess the effects of medium and natural external (environmental) factors on absorption and fluorescence spectra of crude and purified protein extracts from mucus, namely, buffer, redox status, pH, calcium concentration, and salinity. Samples were first dialyzed to seawater, PBS pH 7.4, or 0.05 M Tris-HCl pH 7. The buffer that offered highest fluorescence and reduced intersample variability was selected as vehicle to test the effect of hydrogen peroxide (H_2_O_2_, 2% (*v*/*v*)) and L-dithiothreitol (20 mM DTT) as oxidizing and reducing agents, respectively. The pH of the buffer was then modulated (pH 4, 7, and 9) to determine the most adequate pH following the same criteria. Samples were then subjected to 10- and 100-mM calcium chloride (CaCl_2_) and to 120-mM and 600-mM sodium chloride (NaCl), corresponding to physiological and seawater conditions, respectively. All samples were normalized for the same protein concentration (1 mg·mL^−1^) before assays, which were always performed in triplicate. Replicates consisted of independent mucus samples. All analyses were conducted at room temperature.

Absorption spectra were obtained with an Evolution 300 Spectrophotometer (Thermo Scientific) with 2 nm bandwidth. Emission spectra were analyzed on a Varian Cary Eclipse Fluorescence Spectrophotometer (Agilent, Santa Clara, CA, USA) with 5 nm bandwidth excitation and emission slits in a 3-mm optical path quartz cuvette (HELLMA, Müllheim, Germany). Emission spectra and maxima were determined following excitation at wavelengths determined from the absorption spectra maxima.

### 4.3. Molecular Characterization

#### 4.3.1. Protein Separation

Purified mucus samples 0.05 M Tris-HCl pH 7 were analyzed by sodium dodecyl sulfate-polyacrylamide gel electrophoresis (SDS-PAGE) to separate peptides by molecular mass and isolate the fluorescent band for tandem mass spectrometry analyses (LC-MS/MS). Gels (8 × 9 cm by 0.75 mm thick) were based on the discontinuous system developed by Laemmli [49], as detailed by Hames [50]. In brief: samples were mixed with sampling buffer supplemented with β-mercaptoethanol (1:1), boiled for 2 min (for protein denaturation), and loaded to an acrylamide gel composed of stacking and running gels containing 6% (0.5 M Tris HCl pH 6.8) and 12% (1.5 M Tris-HCl pH 8.8) acrylamide, respectively. The electrophoresis ran between 90–110 in a denaturing buffer containing 25 mM Tris, 192 mM glycine, pH 8.5. The Protein Marker II 11-245 kDa (NZYtech, Lisboa, Portugal) was used as molecular standard. The fluorescent bands were identified and the molecular weight determined in a Gel-Doc 2000 documenting system equipped with a UV transilluminator (BIO-RAD, Hercules, CA, USA). The bands were excised and stored at −80 °C until further analysis. The purified mucus samples were also analyzed by Native PAGE with discontinuous buffer system to verify the persistence of fluorescence under nondenaturing conditions, following Ornstein and Davis [51]. The gels were stained by Coomassie Brilliant Blue R-250 overnight (BIO-RAD) and cleared/fixed with a solution of water:acetic acid:methanol (4:1:5). For better resolution when needed, the gels were also subjected to silver staining according to Rabilloud [52]. In brief, after the gel was sensitized (0.02% sodium thiosulfate, 1 min) and washed in milli-Q water (3 × 20 s), it was incubated (0.1% silver nitrate, 20 min), rinsed again in milli-Q water, and then developed (3% sodium carbonate) until bands were visible or the gel turned yellow. The developing phase was stopped by washing in milli-Q water (20 s); the gel was then fixed in 5% acetic acid (5 min) and stored in 1% acetic acid.

#### 4.3.2. Protein Identification (LC-MS/MS)

After gel digestion with porcine trypsin, the band was analyzed on a NanoLC Ultra 2D separation system (Eksigent, Dublin, OH, USA) coupled to a Triple TOF^TM^ 5600 System mass spectrometer (Sciex, Framingham, MA, USA). The chromatographic separation was performed by the Column ChromXP C18CL (0.3 × 150 mm, 3 μm, 120 Å, Eksigent) at 50 °C. The flow rate was set to 5 μL. min^−1^ and mobile phase A and B were 0.1% formic acid plus 5% (*v*/*v*) DMSO in water and 0.1% (*v*/*v*) formic acid plus 5% (*v*/*v*) DMSO in acetonitrile, respectively. The ionization source (ESI DuoSpray^TM^ Source from Sciex, Framingham, MA, USA) was operated in the positive mode set to an ion spray voltage of 5500 V, with 25 psi for nebulizer gas 1 (GS1) and 25 psi for the curtain gas (CUR). Rolling collision was used with a collision energy spread of 5. Protein and peptide identification were performed using ProteinPilot (Sciex) considering the following parameters: cysteine alkylation by iodoacetamide, digestion by trypsin, gel-based ID as a special factor, and ID focus on biological modification and amino acid substitutions. Positive identification was considered when the identified proteins reached the 1.3 unused score (95% confidence score of Paragon™ Algorithm) and the peptides reached 95% confidence. The interesting candidates were further confirmed by MS/MS spectra evaluation and a minimum sequence tag of 3 amino acids (4 consecutive peaks in the MS/MS spectrum) was required to confirm the identification. The acquired data were contrasted against a customized database containing subsets allocating venom proteins and toxins, Annelida, Conotoxin, Mollusca, and Polychaeta sequences from the UniProtKB (curated) database.

#### 4.3.3. Protein Identification (Multiomics Matching)

The peptide sequences obtained by LC-MS/MS were contrasted with *Eulalia*’s proboscis transcriptome described in previous works [19], deposited in Gene Expression Omnibus (GEO) under accession number GSE143954. The translated cDNAs were contrasted against NCBInr database using Blast [53]. Best hits were selected by number of matching peptides, coverage, and lowest *e*-value.

### 4.4. Histology

Specimens were fixed in 2% (*v*/*v*) glutaraldehyde (in 0.1 mM sodium cacodylate buffer, pH 7.4) during 2 h or in Zenker’s solution (2.5% (*w*/*v*) potassium dichromate, 3% (*w*/*v*) mercury chloride, 1% (*w*/*v*) sodium sulfate, and 5% (*v*/*v*) glacial acetic acid) overnight. Worms were then divided into serial sections, washed in cacodylate buffer (3 × 15 min), dehydrated in a progressive series of ethanol (30–100%), intermediately infiltrated with xylene, and embedded in paraffin (Paraplast) wax. Paraffin-embedded samples were sectioned (5 μm-thickness) with a Jung RM2035 model rotary microtome (Leica Microsystems, Wetzlar, Germany). Histological sections were stained using a tetrachrome stain (TC) and Hematoxylin-Eosin (HE). The tetrachrome protocol is based on Alcian Blue pH 2.5 (AB) for acidic sugars, Periodic Acid/Schiff’s (PAS) for neutral polysaccharides, Weigert’s iron Hematoxylin (WH) for chromatin, and Picric Acid (PA) as counterstain for muscle and cytoplasm. The procedures follow the protocols described by Costa [54].

Other specimens were snap-frozen in liquid nitrogen, divided into six sections, and placed in optimal cutting temperature (OCT) medium in an appropriate tissue mold. The OCT medium with the tissue was then frozen and longitudinal sections of 5–15 μm-thick were cut in a CM3600 XP cryomacrotome (Leica Biosystems) at −20 °C. Sections were transferred to slides with pre-adhesive (Thermo Scientific Superfrost Ultra Plus) and stored at −80 °C until analyses. Slides were then treated with H_2_O_2_ (2% *v*/*v*) and DTT (20 mM) to assess changes to tissue fluorescence. All slides were visualized in a DM 2500 LED model microscope adapted for epifluorescence with an EL6000 light source for mercury short-arc reflector lamps. The microscope was equipped with A, N2.1, and I3 filters (corresponding to blue, red, and green channels, respectively). All equipment was supplied by Leica Microsystems.

### 4.5. Immunohistochemistry

Paraffin and cryopreserved sections were used to analyze immunohistochemically ubiquitin B in *Eulalia* sp., according to Costa [54]. In brief, after deparaffination, both sections were rehydrated in PBS and permeabilized for 15 min with 0.1% Triton X-100 in PBS. Antigen blocking was performed for 30 min in the dark at room temperature, with 2% bovine serum albumin (BSA) in PBS with 0.1% (*v*/*v*) Triton X-100. Slides were then incubated with rabbit antihuman ubiquitin B polyclonal antibody (Invitrogen #PA5-95195) at 1 µg. mL^−1^ in 2% (*w*/*v*) BSA in PBS with 0.1% (*v*/*v*) Triton X-100. Incubation was conducted overnight at 4 °C in a humidity chamber. Slides were afterwards incubated with the secondary antibody incubation for 2 h, the goat antirabbit IGg Alexa Fluor 568 (Invitrogen, Grand Island, NY, USA), with 10 µg. L^−1^ in 2% (*w*/*v*) BSA in PBS with 0.1% (*v*/*v*) Triton X-100. Nuclear staining was performed with DAPI. Slides were visualized as previously described.

### 4.6. Fluorochrome Internalization Assay

Internalization of the purified mucus was assessed through an in vitro assay using the ovarian carcinoma cell line A2780 (purchased from Sigma-Aldrich), taking advantage of the natural fluorescence of the target mucosubstances. Briefly, cells were grown in McCoy’s medium modified as Roswell Park Memorial Institute medium (RPMI 1640) and maintained at 37 °C in a humidified atmosphere with 5% (*v*/*v*) CO_2_. Cells were exposed to the purified extract (1 mg·mL^−1^ in PBS) supplemented and with redox modulators (2% (*v*/*v*) H_2_O_2_ and 20 mM DTT) and controls during 1 h, 3 h, and 6 h, at 37 °C. After the incubation period, cells were washed with PBS and visualized in an Eclipse Ti microscope equipped with a DS-QiMc camera and adapted for epifluorescence (Nikon Instruments, Amsterdam, The Netherlands).

## Data Availability

Bulk data is available as Supplementary Information. Bulk RNA-Seq data is deposited in Gene Expression Omnibus (GEO) under the accession number GSE143954.

## Supplementary Materials

The following supporting information can be downloaded at https://www.mdpi.com/article/10.3390/md20040224/s1, Figure S1: Gel electrophoresis of crude protein extracts purified by ultrafiltration; Table S1: Protein identification by homology matching using BLASTP; Figure S2: Emission spectra of crude mucus samples in different buffers (Seawater, PBS, and Tris-HCl pH 7) and redox treatments (H_2_O_2_ and DTT); Table S2: Averaged peak maximum intensities and respective wavelengths of the emission spectra in crude mucus samples; Figure S3. Emission spectra of purified mucus samples in different buffers (Seawater, PBS, and Tris-HCl pH 7) and redox treatments (H_2_O_2_ and DTT); Table S3. Averaged peak maximum intensities and respective wavelengths of the emission spectra in purified mucus samples; Figure S4: Fluorescence of purified mucus samples in different buffers (Seawater, PBS, and Tris-HCl pH 7) and treated with redox agents (H_2_O_2_ and DTT), under UV-light; Figure S5: Averaged absolute intensity emission spectra of purified mucus samples excited at 285 nm and 330 nm; Figure S6: Emission spectra of reduced purified mucus samples (Tris-HCl plus DTT buffer) at different pH (pH 4, 7, and 9). Table S4: Averaged maximum intensities and respective wavelengths of purified mucus samples (Tris-HCl plus DTT buffer) in the peaks identified in the emission spectra excited at 285 and 330 nm, at different pH levels; Figure S7: Emission spectra of purified mucus samples (Tris-HCl pH 7 plus DTT buffer), with different concentrations of CaCl_2_ (0, 10, and 100 mM); Table S5: Averaged maximum intensities and respective wavelengths of purified mucus samples (Tris-HCl pH 7 buffer) with different CaCl_2_ concentrations (0, 10, and 100 mM); Figure S8: Emission spectra of purified mucus samples (Tris-HCl pH 7 plus DTT buffer), with different concentrations of NaCl (0, 120, and 600 mM); Table S6: Averaged maximum intensities and respective peaks wavelengths for purified mucus samples (Tris-HCl pH 7 plus DTT buffer), with different NaCl concentrations (0, 120, and 600 mM); Table S7: Averaged emission maximum peaks wavelengths, excited at different wavelengths (285, 330, and 374) for purified mucus samples; Figure S9: Internalization assay for purified mucosubstances onto the ovarian cancer cell line A2780.
