# Supplementary material for: Endogenous Fluorescent Proteins in the Mucus of an Intertidal Polychaeta: Clues for Biotechnology
Source: Mar Drugs. 2022 Mar 25;20(4):224. doi: 10.3390/md20040224 (PMC9028460; doi:10.3390/md20040224)
Supplement: Supplementary file 1 [file marinedrugs-20-00224-s001.zip › marinedrugs-1634056-supplementary.pdf]

Endogenous fluorescent proteins in the mucus of an intertidal Polychaeta: Clues for biotechnology

Supplementary Material

Ana P. Rodrigo [1,2,*], Ana Lopes [1], Ricardo Pereira [1], Sandra I. Anjo [3], Bruno Manadas [3], Ana R. Grosso [1,2], Pedro V. Baptista [1,2], Alexandra R. Fernandes [1,2] and Pedro M. Costa [1,2,*]

[1] UCIBIO—Applied Molecular Biosciences Unit, Department of Life Sciences, NOVA School of Science and Technology, Universidade NOVA de Lisboa, 2829-516 Caparica, Portugal; arc.lopes@campus.fct.unl.pt (A.L.); rb.pereira@campus.fct.unl.pt (R.P.); ar.grosso@fct.unl.pt (A.R.G.); pmvb@fct.unl.pt (P.V.B.); ma.fernandes@fct.unl.pt (A.R.F.)

[2] Associate Laboratory i4HB, Institute for Health and Bioeconomy, NOVA School of Science and Technology, Universidade NOVA de Lisboa, 2829-516 Caparica, Portugal

[3] Center for Neuroscience and Cell Biology, University of Coimbra, Parque Tecnológico de Cantanhede, Núcleo 04, Lote 8, 3060-197 Cantanhede, Portugal; sandra.isabel.anjo@gmail.com (S.I.A.); bmanadas@gmail.com (B.M.)

* Correspondence: a.rodrigo@campus.fct.unl.pt (A.P.R.); pmcosta@fct.unl.pt (P.M.C.); Tel.: +351-212-948-300 (NOVA School)

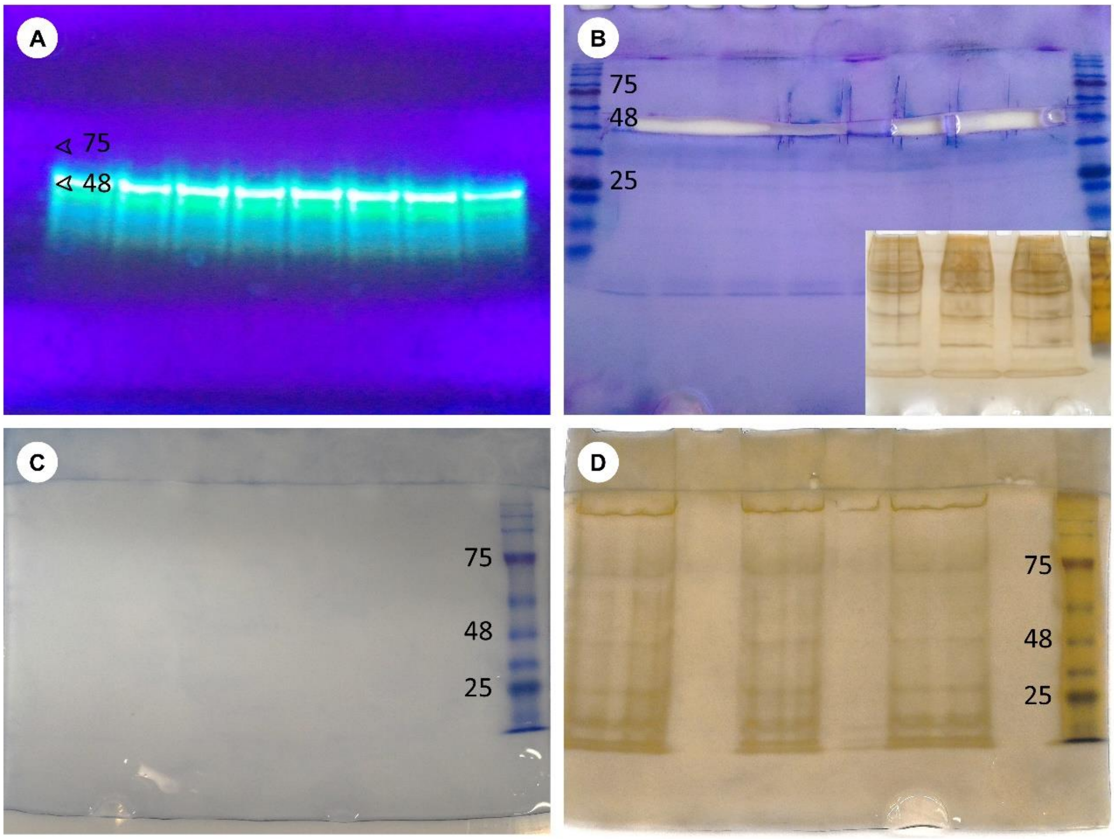

Figure S1. Gel electrophoresis of crude protein extracts purified by ultrafiltration. (**a, b**) The same SDS-PAGE gel showing fluorescent bands under UV-light, followed by excision for MS/MS analysis and staining with Coomassie Blue. *Inset*: SDS-PAGE gel colored by silver staining. (**c, d**) The same native PAGE gel stained by Coomassie blue, followed by silver staining, respectively.

Table S1. Protein identification by homology matching using BLASTP.

Protein	Origin	Conserved domain (Pfam)	Protein match	Query cover	e-value (Blastp)
**Polyubiquitin**	RNA-seq	Ubiquitin-like domain	Polyubiquitin-B (*Nematostella vectensis*)	100%	1×10^{-88}
	MS-MS	Ubiquitin-like domain	Polyubiquitin-C (*Nematostella vectensis*)	100%	9×10^{-10}
**Peroxiredoxin**	RNA-seq	Peroxiredoxin family, 2-Cys PRX subfamily	Peroxiredoxin-1 (*Theropithecus gelada*)	94%	2×10^{-147}
	MS-MS	Tryparedoxin peroxidase	Peroxiredoxin-1-like (*Dendronephthya gigantea*)	100%	1×10^{-04}
**14-3-3**	RNA-seq	14-3-3 superfamily	14-3-3 zeta (*Pristionchus pacificus*)	95%	3×10^{-121}
	MS-MS	-	14-3-3 protein beta/alpha-A (*Stylophora pistillata*)	100%	6×10^{-04}

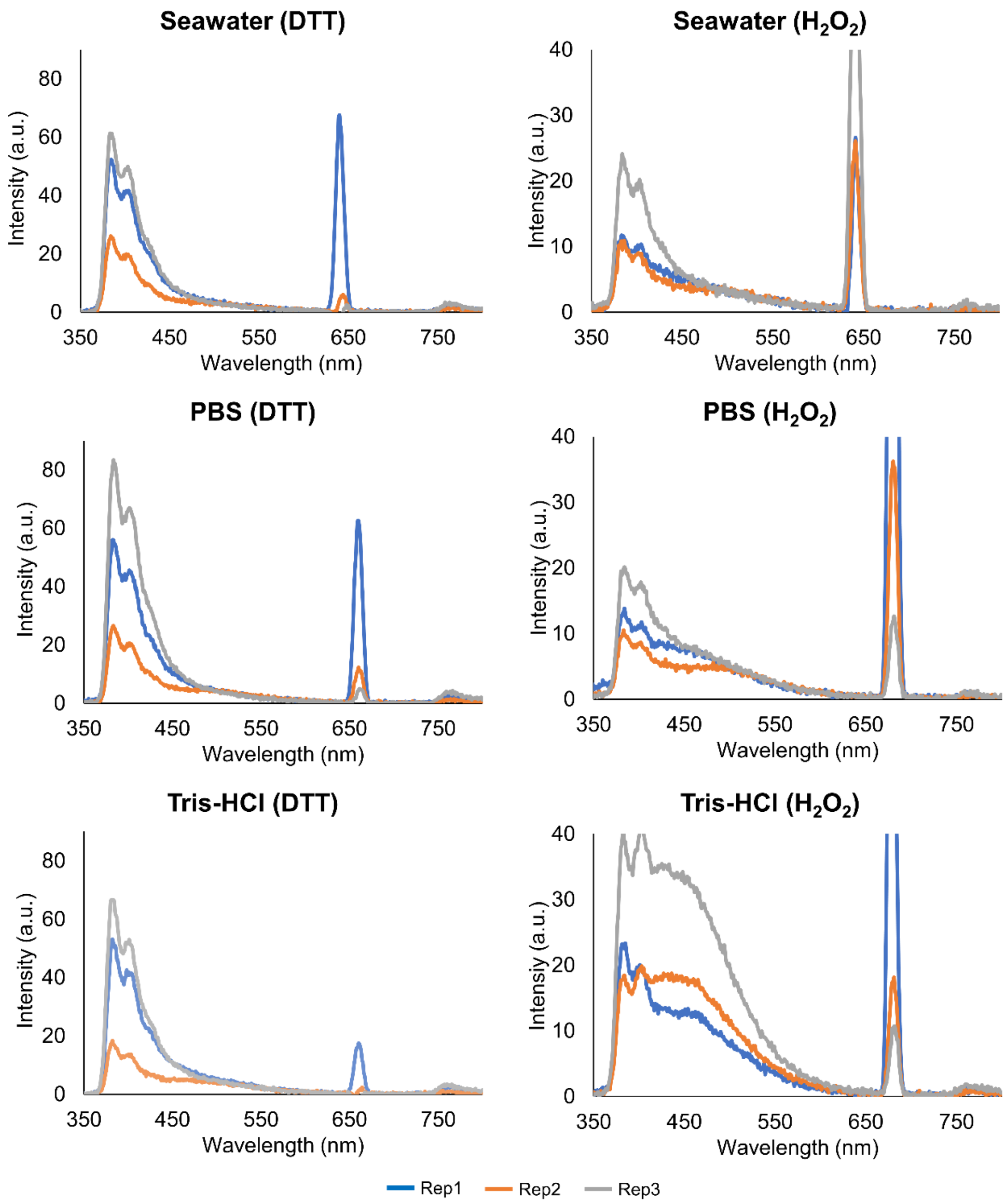

Figure S2. Emission spectra of crude mucus samples in different buffers (Seawater, PBS and Tris-HCl, pH 7) and redox treatments (H_2O_2 and DTT). Samples were excited at 320-340 nm. Rep1 to Rep3 indicate the three independent replicates. Concentration of extracts was normalised to 1 mg total protein. mL^{-1}.

Table S2. Averaged peak maximum intensities and respective wavelengths of the emission spectra in crude mucus samples. Samples were excited at 260 and 320-340 nm for oxidised (H_2O_2) and reduced samples (DTT). Experiments were done in triplicate with independent extracts.

Buffer	Excitation (nm)	Emission max (nm)	Intensity max (a.u.)	SD (a.u.)
Seawater + H_2O_2	255	384	8.36	2.66
		400	7.37	2.11
	340	383	15.08	4.71
		403	13.07	3.95
PBS + H_2O_2	250	385	7.2	2.9
		401	6.18	2.16
	340	384	15.25	3.76
		402	12.97	3.73
Tris-HCl + H_2O_2	250	384	10.10	3.96
		401	8.63	3.44
	340	383	27.23	9.33
		403	26.66	9.83
Seawater + DTT	255	384	55.74	15.49
		402	44.72	12.76
	340	385	44.06	15.67
		402	35.84	12.89
PBS + DTT	260	384	70.36	26.65
		404	55.89	21.75
	330	384	57.04	23.1
		403	45.76	18.86
Tris-HCl + DTT	260	384	50.21	21.31
		403	39.09	17.02
	330	383	47.83	20.99
		402	37.69	17.11

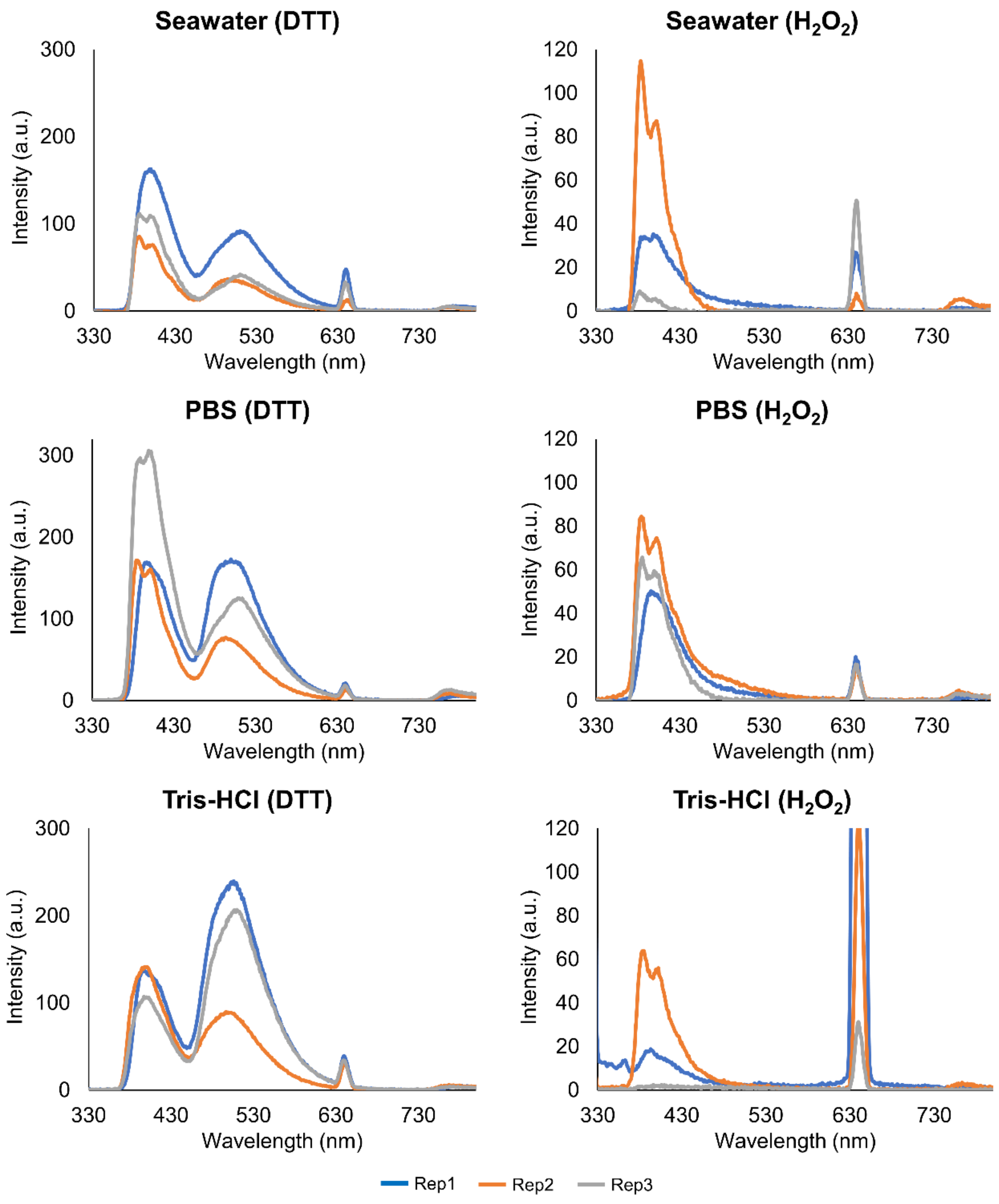

Figure S3. Emission spectra of purified mucus samples in different buffers (Seawater, PBS and Tris-HCl, pH 7) and redox treatments (H_2O_2 and DTT). Samples were excited at 320 nm. Rep1 to Rep3 indicate three the independent replicates. Concentration of extracts was normalised to 1 mg total protein. mL^{-1}.

Table S3. Averaged peak maximum intensities and respective wavelengths of the emission spectra in purified mucus samples. Samples were excited at 286 and 320 nm for oxidised samples (H_2O_2) and at 260, 286 and 320 nm for reduced samples (DTT). Experiments were done in triplicate with independent extracts.

Buffer	Excitation (nm)	Emission max (nm)	Intensity max (a.u.)	SD (a.u.)
Seawater + H_2O_2	286	382	67.69	50.74
		400	55.53	38.22
	320	382	52.36	45.31
		400	42.15	33.65
PBS + H_2O_2	286	386	60.59	13.23
		399	59.18	6.44
	320	385	61.43	18.97
		400	60.56	10.44
Tris-HCl + H_2O_2	286	383	31.94	32.79
		398	28.08	26.79
	320	384	26.94	26.78
		399	24.50	22.48
Seawater + DTT	260	402	199.93	69.09
		509	54.71	24.31
	286	399	170.84	58.94
		510	44.34	18.19
	320	398	115.92	35.74
		509	55.99	25.76
PBS + DTT	260	399	328.62	128.22
		511	100.54	32.74
	286	399	290.00	108.44
		510	77.64	22.00
	320	398	210.76	67.13
		509	120.46	40.89
Tris-HCl + DTT	260	398	204.66	18.78
		508	174.26	29.23
	286	398	185.33	20.28
		509	133.98	25.82
	320	396	128.31	15.14
		505	177.49	65.43

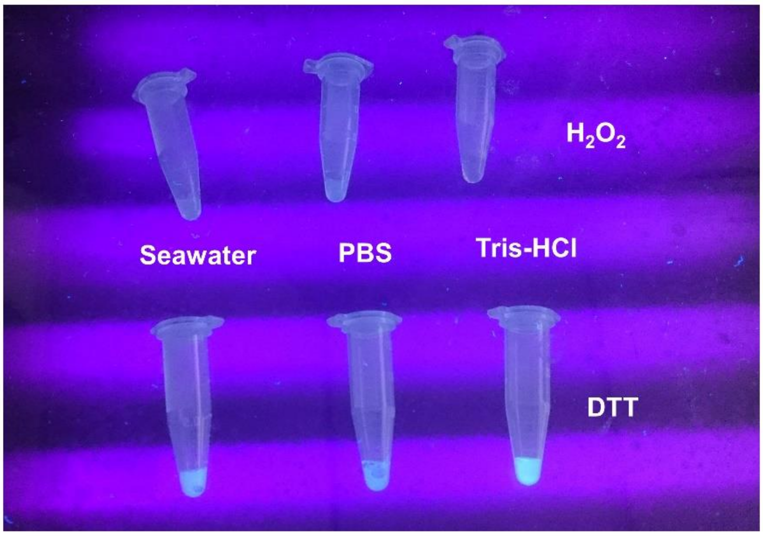

Figure S4. Fluorescence of purified mucus samples in different buffers (Seawater, PBS and Tris-HCl pH7) and treated with redox agents (H_2O_2 and DTT), under UV-light. The blue-greenish fluorescence is notorious in all samples treated with DTT.

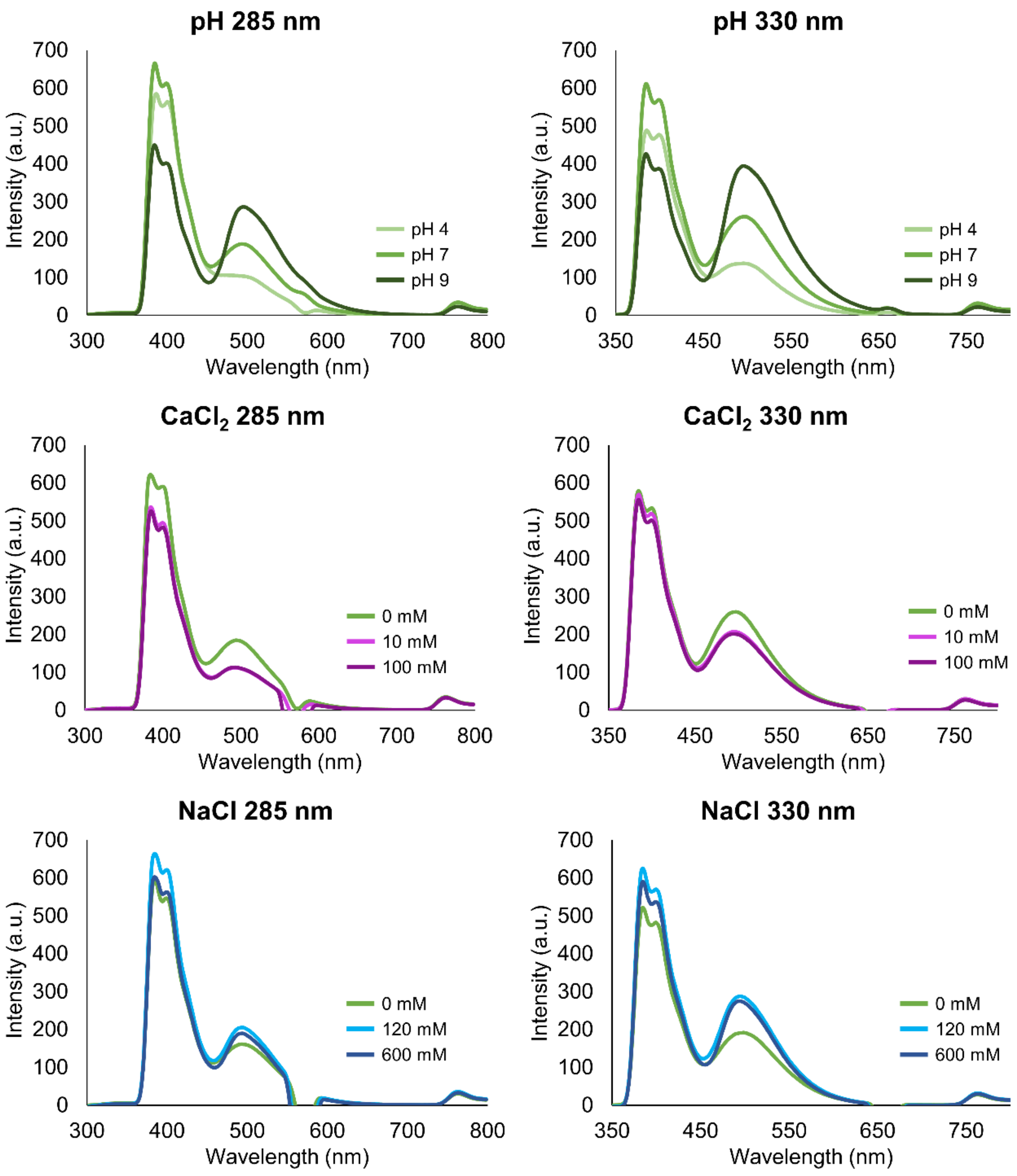

Figure S5. Averaged absolute intensity emission spectra of purified mucus samples excited at 285 nm and 330 nm. Samples were kept in Tris-HCl, 20 mM DTT buffer and modulated according to: pH (4, 7 and 9), calcium chloride (0, 10 and 100 mM $CaCl_2$), sodium chloride (0, 120 and 600 mM NaCl). Experiments were done in triplicate with independent extracts. Concentration of extracts was normalised to 1 mg total protein. mL^{-1}.

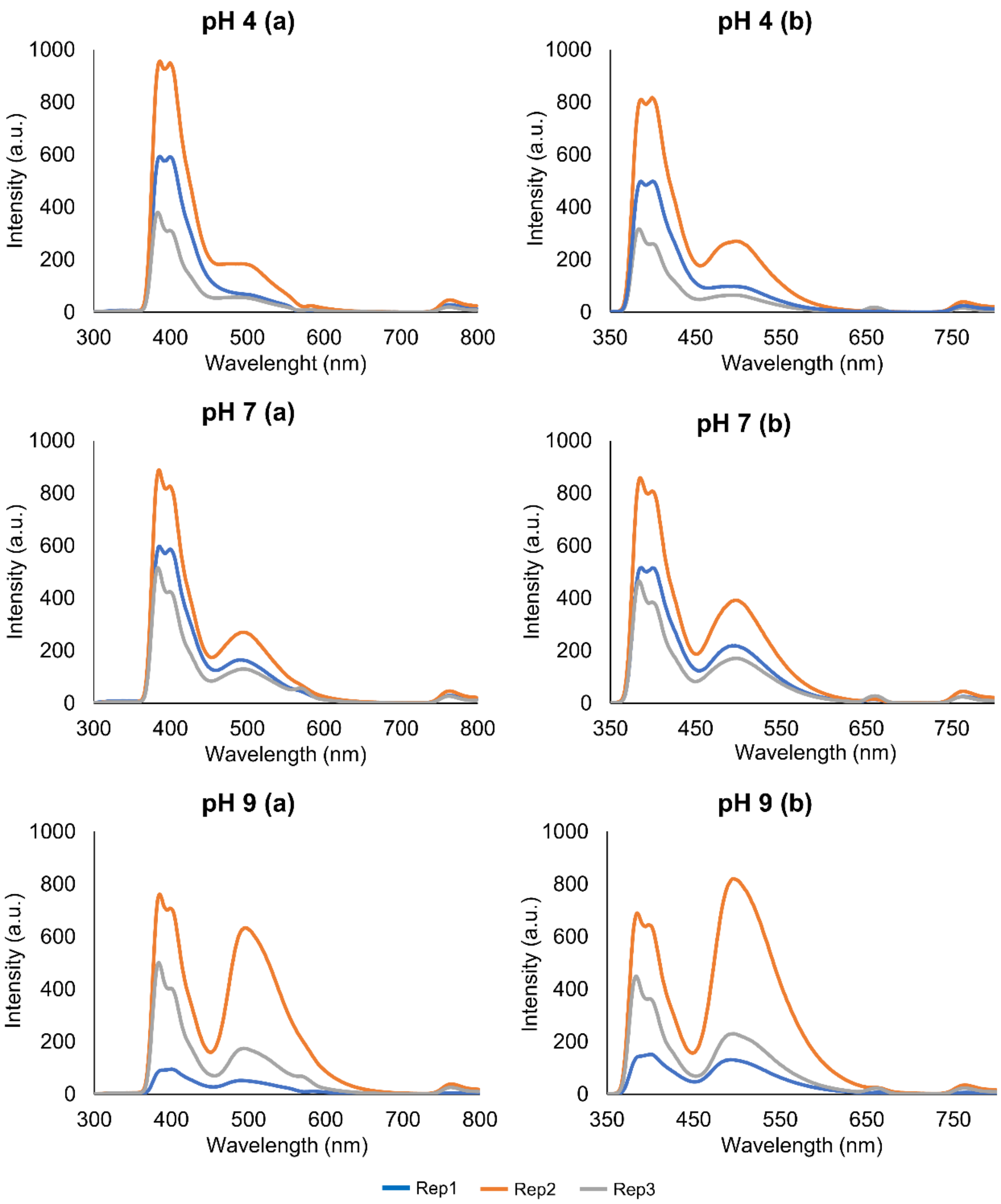

Figure S6. Emission spectra of reduced purified mucus samples (Tris-HCl plus DTT buffer), at different pH (pH 4, 7 and 9). Samples were excited at 285 (a) and 330 nm (b). Rep1 to Rep3 indicate three the independent replicates. Concentration of extracts was normalised to 1 mg total protein. mL^{-1}.

Table S4. Averaged maximum intensities and respective wavelengths of purified mucus samples (Tris-HCl plus DTT buffer) in the peaks identified in the emission spectra excited at 285 and 330 nm, at different pH levels. Experiments were done in triplicate with independent extracts.

Buffer	Excitation (nm)	Emission max (nm)	Intensity max (a.u.)	SD (a.u.)
Tris-HCl pH 4	285	385	584.32	261.50
		399	563.43	277.27
		491	103.90	57.76
	330	385	487.95	225.89
		399	476.81	243.76
		496	137.17	94.42
Tris-HCl pH 7	285	384	665.07	159.75
		399	563.43	277.27
		493	188.51	59.35
	330	385	611.53	176.25
		399	569.30	176.88
		496	260.59	94.94
Tris-HCl pH 9	285	384	449.54	276.42
		399	563.43	277.27
		495	286.66	250.73
	330	385	425.88	224.15
		399	386.75	201.95
		496	394.28	304.50

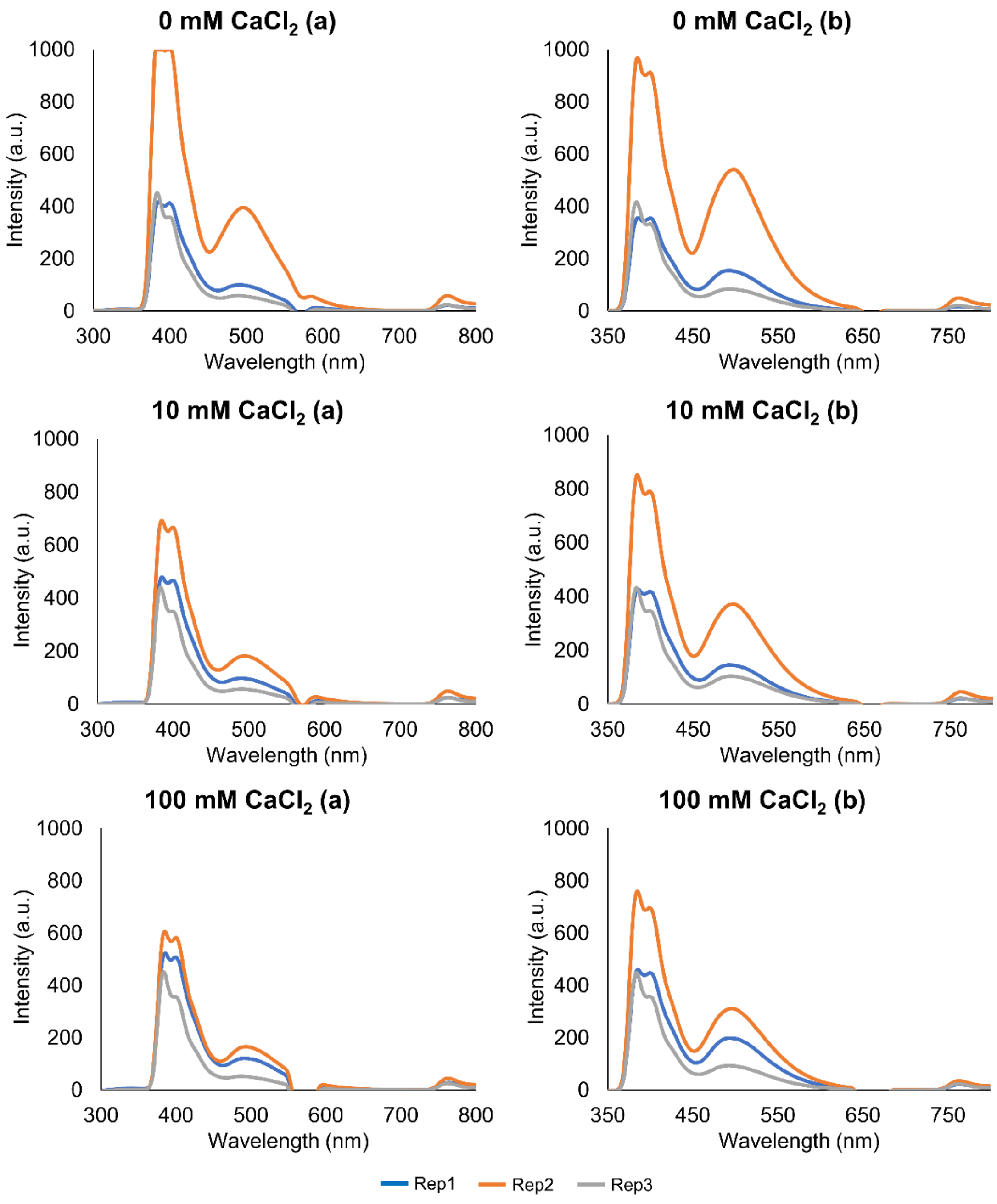

Figure S7. Emission spectra of purified mucus samples (Tris-HCl pH 7 plus DTT buffer), with different concentrations of $CaCl_2$ (0, 10 and 100 mM). Rep1 to Rep3 indicate three the independent replicates. Concentration of extracts was normalised to 1 mg total protein. mL^{-1}. Samples were excited 285 (a) and 330 nm (b).

Table S5. Averaged maximum intensities and respective wavelengths of purified mucus samples (Tris-HCl pH 7 buffer) with different $CaCl_2$ concentrations (0, 10 and 100 mM). Samples were excited at 285 and 330 nm. Experiments were done in triplicate with independent extracts.

Buffer	Excitation (nm)	Emission max (nm)	Intensity max (a.u.)	SD (a.u.)
$CaCl_2$ 0 mM	285	384	621.73	267.38
		399	590.24	290.15
		494	184.65	150.16
	330	385	578.66	276.09
		400	533.81	268.05
		497	259.66	201.36
$CaCl_2$ 10 mM	285	384	535.60	110.46
		399	494.40	130.45
		491	111.97	51.41
	330	385	568.89	200.12
		399	519.08	194.72
		497	206.77	118.04
$CaCl_2$ 100 mM	285	385	525.30	64.87
		400	482.79	93.96
		492	113.14	46.99
	330	385	555.29	145.34
		399	501.10	143.44
		494	201.26	89.01

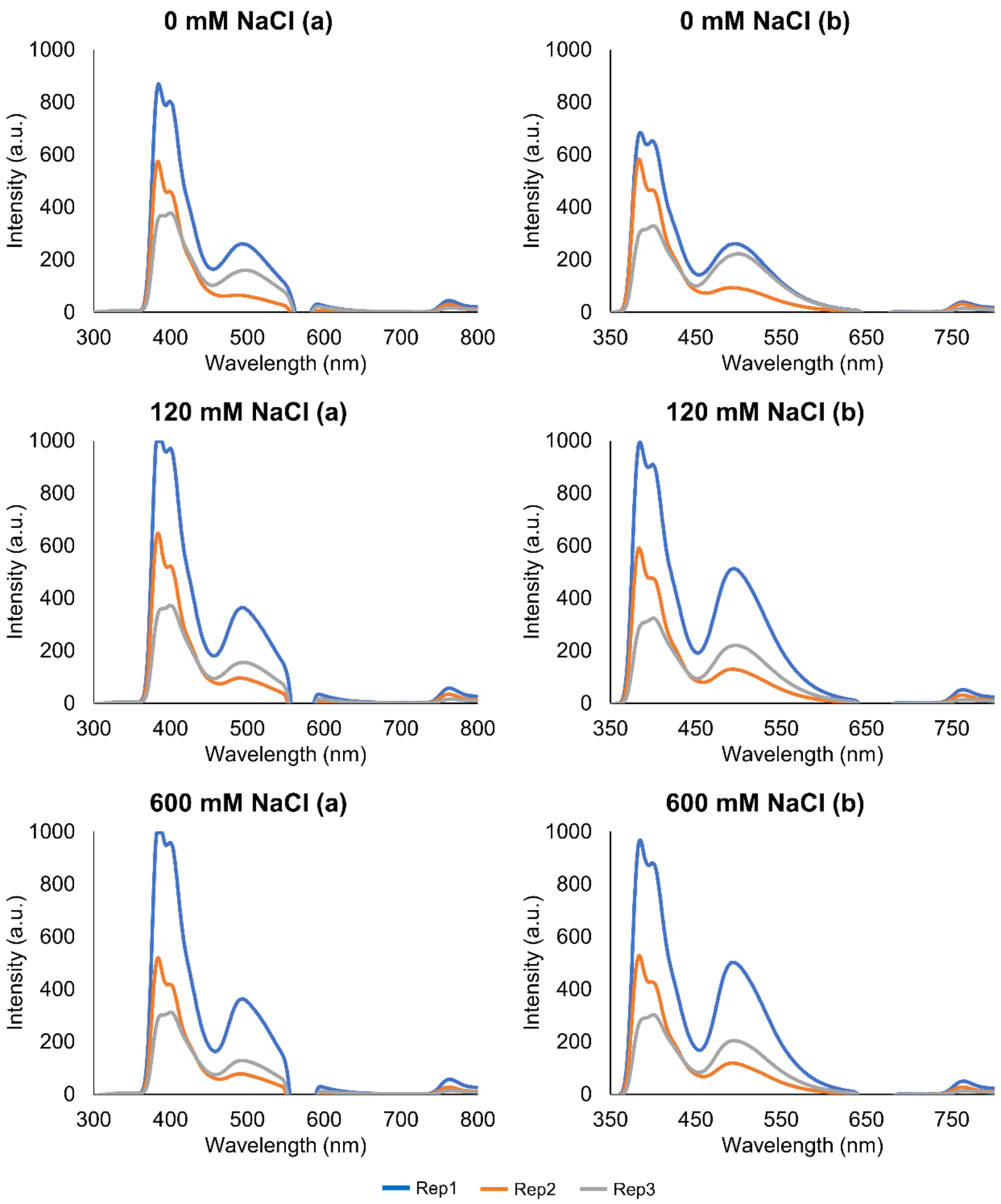

Figure S8. Emission spectra of purified mucus samples (Tris-HCl pH 7 plus DTT buffer), with different concentrations of NaCl (0, 120 and 600 mM). Rep1 to Rep3 indicate three the independent replicates. Concentration of extracts was normalised to 1 mg total protein. mL^{-1}. Samples were excited at 285 (a) and 330 nm (b).

Table S6. Averaged maximum intensities and respective peaks wavelengths for purified mucus samples (Tris-HCl pH 7 plus DTT buffer), with different NaCl concentrations (0, 120 and 600 mM). Samples were excited at 285 and 330 nm. Experiments were done in triplicate with independent extracts.

Buffer	Excitation (nm)	Emission max (nm)	Intensity max (a.u.)	SD (a.u.)
NaCl 0 mM	285	384	600.12	209.76
		399	546.57	184.32
		493	161.31	80.28
	330	385	521.70	158.95
		399	482.19	132.77
		498	191.62	71.40
NaCl 120 mM	285	384	663.70	266.21
		399	621.27	254.07
		493	205.23	115.14
	330	385	624.94	286.40
		400	569.84	247.67
		495	287.85	163.43
NaCl 600 mM	285	384	602.35	294.37
		399	563.08	283.23
		494	189.59	124.79
	330	385	590.16	285.23
		399	536.71	248.75
		495	274.86	164.49

Table S7. Averaged emission maximum peaks wavelengths, excited at different wavelengths (285, 330 and 374) for purified mucus samples. Samples were in different buffers treated with DTT (Seawater, PBS and Tris-HCl pH 7), different pHs (pH 4, 7 and 9 in Tris-HCl 20 mM DTT buffer), different $CaCl_2$ concentrations (0, 10 and 100 mM in Tris-HCl 20 mM DTT pH 7) and different NaCl concentrations (0, 120 and 600 mM in Tris-HCl 20 mM DTT pH 7). Experiments were done in triplicate with independent extracts.

Fluorescence emission maxima wavelengths (nm)

		Excitation Wavelength		
		**285 nm**	**330 nm**	**374 nm**
**Buffer**	Seawater	513	507	509
**(DTT)**	PBS	510	504	507
	Tris-HCl	509	505	507
**pH**	4	492	496	486
	7	493	496	493
	9	495	496	495
**$CaCl_2$**	0 mM	494	497	495
	10 mM	491	497	494
	100 mM	493	496	490
**NaCl**	0 mM	493	499	493
	10 mM	493	495	494
	100 mM	494	495	494

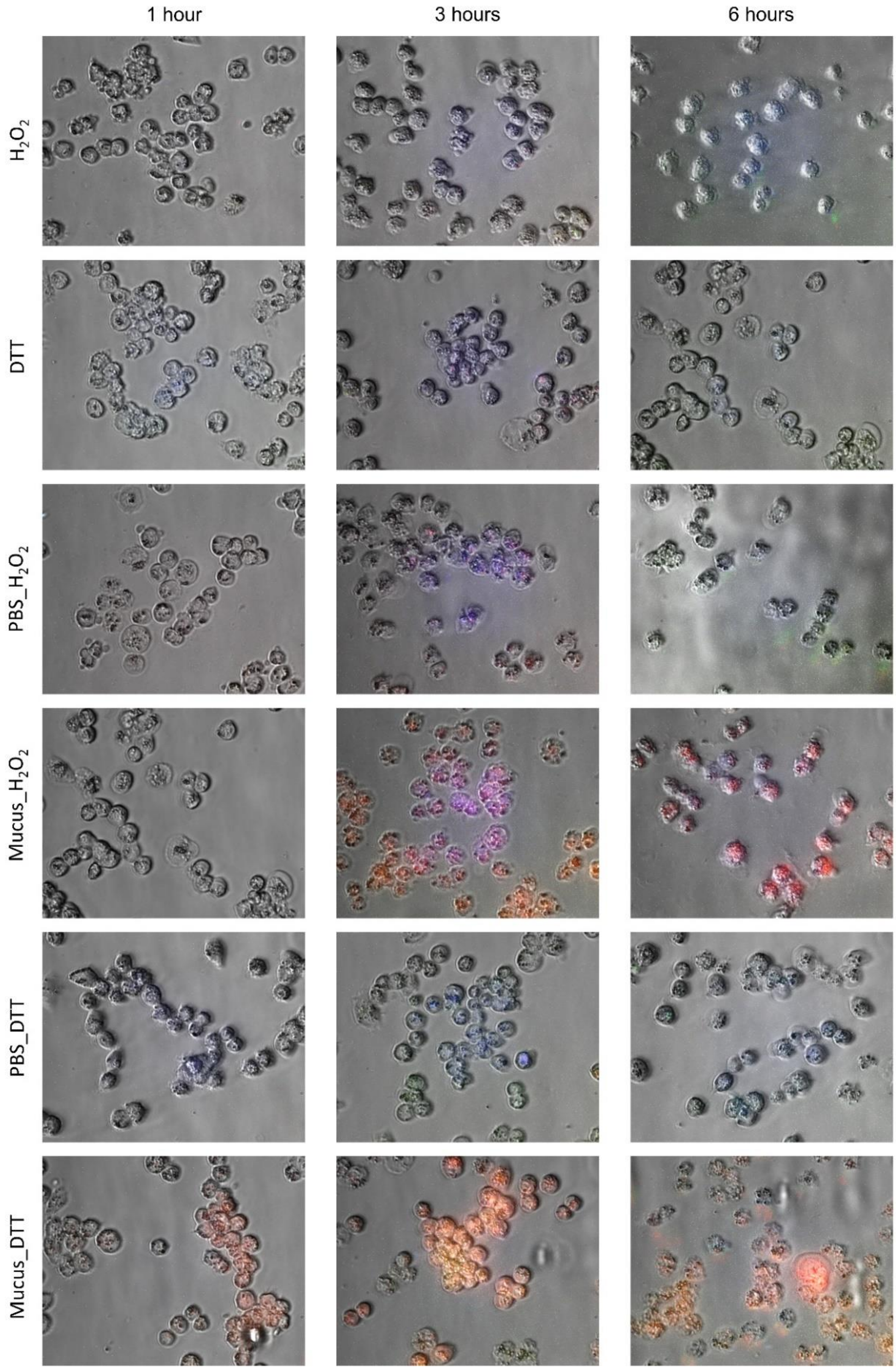

Figure S9. Internalisation assay for purified mucosubstances onto the ovarian cancer cell line A2780. Cells were incubated during 1 h, 3 h and 6 h with purified mucus extract prepared with PBS (pH 7.4) and the respective control (PBS). Either experimental condition was subjected to

reduced or oxidizing agents (DTT and H_2O_2). Composite images were produced by overlapping red, green and blue channels with brightfield images.
